# Built-in buoyancy enables efficient water energy harvesting

**DOI:** 10.1093/nsr/nwaf514

**Published:** 2025-11-25

**Authors:** Lili Wang, Zuankai Wang

**Affiliations:** Department of Mechanical Engineering, The Hong Kong Polytechnic University, China; Department of Mechanical Engineering, The Hong Kong Polytechnic University, China

Harnessing water for power generation is crucial for tackling the global energy crisis. Now, a buoyancy-enabled droplet electricity generator offers a renewable and clean alternative pathway for water energy harvesting.

Recent advances in water energy harvesting techniques, including hydrovoltaic technology, triboelectric nanogenerators and reverse electrodialysis, have resulted in a renaissance in this area of research, revolutionizing our understanding of the fundamentals underpinning power generation [1–3]. Of particular importance is the invention of transistor-like droplet electricity generators that can dramatically enhance energy conversion efficiency through a novel bulk effect [4,5], in contrast to conventional interfacial effects. Central to this technology is the ingenious design of electrical heterogeneity, where a reconstructed electric field is aligned with the fluid field, thus liberating the stored electrostatic energy in the dielectric material. Now writing in *National Science Review*, Deng and colleagues leverage this transistor-like architecture in designing a floating droplet electricity generator that harnesses built-in buoyancy, providing a lighter and more economical solution for water energy harvesting in land-free scenarios [6].

**Figure 1.**
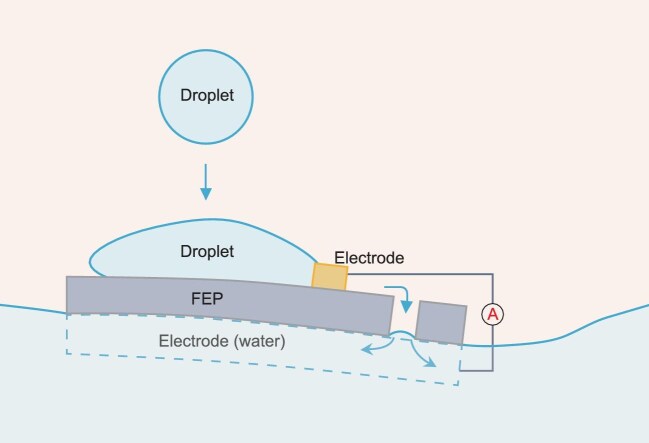
Schematic of a floating droplet electricity generator that employs water as both the bottom electrode and the supporting substrate, enabling cost-efficient water energy harvesting in land-free scenarios.

Covering two-thirds of the earth’s surface, water is endowed with inherent advantages such as high flowability, kinetic energy and surface energy. Here, building upon these properties of water, Deng and colleagues constructed a transistor-like droplet electricity generator on water with built-in buoyancy. In this design, FEP (fluorinated ethylene propylene) film serves as the dielectric layer, with a metal wire acting as the top electrode, while water functions concurrently as both the bottom electrode and the substrate, as shown in Fig. 1. This floating device exhibits outstanding output performance while maintaining excellent durability under various environmental conditions. The boosted output performance originates from a reversible charge transfer mechanism via a bulk effect [4], which relies on the charges stored in the dielectric material, instead of just interfacial contact electrification. In such a mechanism, the reversible and efficient electricity generation requires timely shedding of impinging droplets from the device surface. To this end, Deng and colleagues engineered a drain hole, with a characteristic length of 3 mm (slightly larger than the capillary length of water), on the dielectric material. This drain hole enables effective droplet removal from the device surface, thus avoiding unwanted formation of water film.

Despite the remarkable performance of this floating droplet electricity generator, further optimization would realize its full potential. To enhance the average power density, one option would be to improve the working, or impinging, frequency by minimizing the droplet’s contact time with the deformable (hence dissipation-sensitive) water substrate, as well as its sliding time through the drain hole. This can be achieved by engineering topological structures or wettability gradients mimicking lotus leaves on the device surface for more efficient droplet removal and transport [7]. Moreover, long-term self-cleaning and power generation stability of this floating device under harsh environments (e.g. high humidity and low temperature) can be reinforced by incorporating a slippery lubricant-impregnated porous surface (SLIPS) [8] inspired by the pitcher plant. This SLIPS enables liquid–liquid contact between the droplet and the device surface, thereby effectively eliminating contact line pinning and biofilm formation in wet conditions. From a sustainability perspective, the entire device can be constructed from natural or biodegradable alternatives [9]. For practical deployment in open water areas, such a floating device necessitates an anchoring system to bolster position stability and can be further adapted for hybrid energy harvesting (e.g. from waves and tides) via rational interface design and material optimization [5].

Overall, although the idea of using fluidic water electrodes for electricity generation is not new [10], the work by Deng and colleagues represents one step forward towards efficient water energy harvesting in land-scarce regions. Beyond this work, even though many questions (e.g. the upper limit in energy conversion efficiency) in this field remain unanswered, the underlying principle of droplet electricity generators carries significant implications for the development of new energy harvesting technologies that utilize water and other resources.
